# Scientific Items

**Published:** 1884-07-20

**Authors:** 


### SCIENTIFIC ITEMS.

**Victor Hugo on Sewage Irrigation.**—More than twenty years ago, Victor Hugo wrote as follows in Les Miserables:

"The earth impoverished by the sea. Paris throws twenty-five million dollars a year into the sea. And this without metaphor. How, and in what manner? Day and night. With what object? Without any object. With what thought? Without thinking. For what reason? For nothing. By means of what organ? By means of its intestine. What is its intestine? Its sewer. * * * Science, after long experiment, now knows that the most fertilizing and the most effective of manures is that of man. The Chinese, we must say to our shame, knew it before us. No Chinese peasant, Eckeberg tells us, goes to the city without carrying back at the two ends of his bamboos two buckets full of what we call filth. Thanks to human fertilization, the earth in China is still as young as in the days of Abraham. Chinese wheat yields a hundred and twenty fold. There is no guano comparable in fertility to the detritus of the capital. A great city is the most powerful of stercoraries. To employ the city to enrich the plains would be a sure success. If our gold is filth, on the other hand our filth is gold. What is done with this filth? Gold! It is swept into the abyss. We fit our convoys of ships, at great expense, to gather up at the south pole the droppings of petrels and penguins; and the incalculable elements of wealth which we have under our own hands we send to the sea. All the human and animal manure which the world loses, restored to the land instead of being thrown into the water, would suffice to nourish the world. These heaps of garbage at the corners of the stone blocks, these tumbrils of mire jolting the streets at night, these horrid scavengers' carts, these fetid streams of subterranean slime which the pavements hide from you—do you know what all this is? It is the flowering meadow: it is the green grass; it is marjoram and thyme and sage; it is game; it is cattle; it is the satisfied low of huge oxen at evening; it is perfumed hay, it is golden corn; it is bread on your table; it is warm blood in your veins; it is health; it is joy; it is life. * * * Statistics show that France, alone, makes a liquidation of a hundred millions every year into the Atlantic Ocean from the mouths of her rivers. * * * The cleverness of man is such that he prefers to throw this hundred millions into the gutter. It is the very substance of the people which is carried away—here drop by drop, there in floods—by the wretched vomiting of our sewers into the rivers, and the gigantic collection of our rivers into the ocean. Each hiccough of our cloaca costs us one thousand francs. From this come two results—the land impoverished and the water infected. Hunger rising from the furrow, and disease rising from the river. * * * A system of elementary drainage as simple as the lungs of a man, and which is already in full operation in several villages in England, would suffice to bring into our cities the pure water of the fields and send back into our fields the rich water of the cities. And this easy see-saw, the simplest in the world, would retain in our possession the hundred millions thrown away. The present system does harm in endeavoring to do good. The intention is good; the result is bad."—*Pop. Sciance News.*

**Ericsson's New Solar Motor.**—We have reported in Science News, from time to time, sundry ingenious attempts to utilize the enormous motive-power that is wasted in the sunshine falling upon the earth. That this source of power will hereafter come to be used on an extensive scale is not to be doubted, and inventors may well be giving attention to the means of doing this economically. Mr. John Ericsson, who has long been experimenting in this direction, has recently devised one of the most promising solar motors yet made. It consists of a steam engine, supplied with steam from a small boiler close by, which is fixed in the focus of an immense parabolic mirror. The mirror is cheaply made, consisting merely of slips of silvered glass, each three inches wide and twenty-six inches long, set in a frame so shaped as to concentrate the rays reflected from them into a single narrow line. In this line is set the boiler, consisting of a tube, which serves as a brace to the frame of the mirror. The mirrors, when properly exposed to the sunlight, serve to intercept a beam of about one hundred and fifty square feet in section, and to concentrate these rays by reflection upon an area of about eight and one-half square feet, increasing the heat nearly twenty times, and readily boiling the water contained in the tube in the focus. The steam so generated is conveyed to the cylinder of the engine by a flexible tube, and the mirror-frame is pivoted in such a way that it can be moved to follow the sun's course through the sky. In the model engine constructed for experiment, the pressure of steam was easily kept up to thirty-five pounds to the square inch; and the average speed of the engine, working with a six-inch cylinder and eight inches stroke, was one hundred and twenty revolutions a minute.—*Pop. Science News.*

**Dreams.**—The British Medical Journal records some interesting experiments on this subject, made by M. G. Delaunay. According to psychologists, dreams are generally illogical and absurd. M. Delaunay, however, by covering his forehead with a layer of cotton wool, rendered, at will, his dreams healthy and intelligent. His experience is that dreams while lying on the back are sensuous, agitated and erotic; on the right side, changeable, full of exaggeration, absurd, and relate to old recollections; on the left side, intelligent, reasonable and relate to recent occurrences. People often talk during the latter. The author considers that dreams, intelligent or otherwise, erotic or sober, can be induced by causing variations in the cerebral circulation and the nutrition of the various regions of the brain, either by the elevation of the cranial temperature or by decubitus. They, therefore, form an interesting field for psychological research.—*Medical and Surgical Reporter.*

